# Mechanism of Antiviral Activities of Nanoviricide's Platform Technology based Biopolymer (NV-CoV-2)

**DOI:** 10.3934/publichealth.2022028

**Published:** 2022-04-12

**Authors:** Ashok Chakraborty, Anil Diwan, Vinod Arora, Yogesh Thakur, Vijetha Chiniga, Jay Tatake, Rajesh Pandey, Preetam Holkar, Neelam Holkar, Bethany Pond

**Affiliations:** 1 Department of Biochemistry, AllExcel, Inc., 118, Wood Street, Ste 201, West Haven, CT 06516, USA; 2 Department of Virology, NanoViricides, Inc., 1, Controls Dr. Shelton, CT 06484, USA

**Keywords:** biopolymer, NV-CoV-2, SARS-CoV-2, COVID-19, antiviral mechanism

## Abstract

NV-CoV-2 is a nanoviricide that is covalently bonded with polyethylene glycol (PEG) and alkyl pendants. This molecular design is used to attack many strains of coronaviruses in a broad-spectrum manner. The ligand works by competitive inhibition and binds to the same site on the S-protein of SARS-CoV that attaches to the cognate cellular receptor, ACE2. This prevents SARS-CoV from binding and infecting the cell. NV-CoV-2 is designed to bind to the free virion particles at multiple points encapsulate the virus and disable its ability to infect the cells. The multi-point binding interaction, like a nano-velcro-tape, may lead to lipid-lipid fusion of the alkyl chains in the nanoviricide micelle with the lipid envelope of the virus. The virus becomes dismantled to a capsid form before the host immune system becomes involved. This putative mechanism is orthogonal to many other anti-coronavirus agents in development. Thus, it maybe possible to produce a stronger antiviral effect when combining NV-CoV-2 therapy with other anti-coronavirus therapies such as Remdesivir (RDV). NV-CoV-2 can encapsulate other antiviral compounds as well. In this study, RDV was encapsulated and protected from serum-mediated degradation *in vivo*. As a result, RDV was available for a longer period of time to interact with RNA polymerase and inhibit.

## 1. Introduction

**Figure 1.**
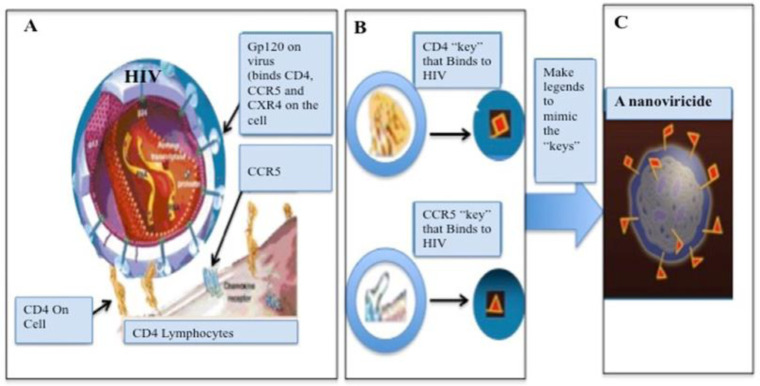
Novel platform technology: A nanoviricide is a cell mimic. A, B: HIV particle is binding to a cell via CD4 and a co-receptor. C: A nanoviricide “looks like” a human cell to the virus.

Nanoviricide^®^ is a platform technology based biopolymer that is used as a broad-spectrum antiviral compound [1]. The flexible polymer backbone is comprised of a polyethylene glycol (PEG) and alkyl pendants. PEG forms the hydrophilic shell and imparts non-immunogenicity. In fact, a familiar technique used to minimize immunogenicity of proteins and antibodies is known as PEGylation. The alkyl chains float together to make a flexible core, like an immobilized oil droplet. The resulting materials are polymeric surfactants that form stable micelles and not dynamic micelles that liposomes form. Chemical groups are uniformly distributed along the polymer chain, which allow attachment of virus-specific ligands like chemical moieties, peptides, antibody fragments or other proteins (Figure 1).

Nanoviricides are large enough for a virus particle to latch onto it, yet small enough to be circulated in the body. A nanoviricide wraps around the virus particle and encapsulates it, preventing the virus's ability to enter a cell. The viral resistance to the nanoviricide drug is unlikely because it still will bind to the same cell surface receptors, even if the virus mutates.

NV-CoV-2 is a nanoviricide that is recently being developed to treat the SARS-CoV-2 infection. NV-CoV-2 is an off-white, odorless material. At room temperature the material is a waxy, non-crystalline semi-solid that tastes slightly sour [2] (https://www.genengnews.com/covid-19-candidates/nanoviricides/).

Proof-of-Concept studies were completed through both *in vivo* and *in vitro* studies. The human coronavirus, CoV-NL63 was used in the virus-infected cell culture and rats [3]–[8]. Similarly to SARS-CoV-2, CoV-NL63 also binds to the angiotensin-converting enzyme 2 (ACE2) in order to enter into the host cells [6],[7]. Therefore, CoV-NL63 is an appropriate model virus for the proof-of-concept studies for both *in vitro* and *in vivo* [3]–[8].

## 2. Mechanism of action

### 2.1. The broad spectrum of the antiviral activity of a nanoviricide

**Figure 2.**
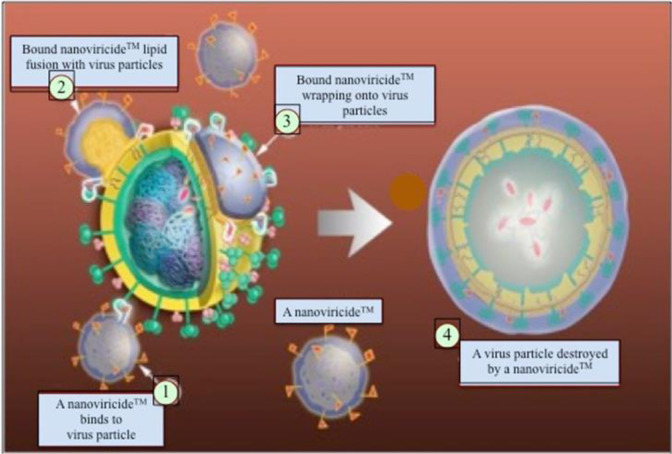
The graphic representation of the mechanism of action when a *Nanoviricide* binds and inactivates the viruses. Step 1: A *nanoviricide™* binds to virus particle; Step 2: Lipid fusion of bound *nanoviricide™* with the virus particle; Step 3: Bound *nanoviricide™* encapsulates the virus particle; Step 4: The virus particle is destroyed by a *nanoviricide™*.

A nanoviricide is composed of a number of antiviral small chemical ligands that are covalently attached to a micelle-forming polymeric chain. The polymeric chain is a homopolymer of a monomer that is covalently bonded to polyethylene glycol (PEG), a connecting group and alkyl pendants. Self-assembly of the polymeric chain will occur in an aqueous solution where the PEG shell surrounds the hydrophobic alkyl core. This particular ligand is designed through molecular modeling to bind to the S-protein of SARS-CoV-1 and prevent the virus from binding to the cognate cellular receptor, ACE2. Therefore, the nanoviricide, NV-CoV-2 was designed to prevent coronaviruses such as SARS-CoV-1, SARS-CoV-2 and NL63 from binding to the ACE2 receptor. In addition, NV-CoV-2 has similar activity against the human coronavirus 229E (hCoV-229E), which uses Aminopeptidase N (APN) as its cellular receptor instead of ACE2 [7]. There are certain conserved elements in the quaternary structures in both peptidases ACE2 and APN that may explain why NV-CoV-2 works similarly on both [8]. The broad-spectrum activity of NV-CoV-2 working against both hCoV-NL63 and hCoV-229E indicates that it may continue to stay active against a number of coronaviruses, even after it mutates [5]–[11]. This proposed mechanism of action is shown graphically in Figure 2.

### 2.2. Encapsulation of the virus leads to disintegration

NV-CoV-2 is designed to bind to the free virion particles at different places through receptor-ligand interaction and encapsulate the virus. Encapsulation of the virus disables its ability to infect the cells. Similarly to “nano-velcro-tape”, this multiple binding interaction may lead to a lipid-lipid fusion of the alkyl chains in the nanoviricide micelle with the lipid envelope of the virus. This dismantles the virus to a capsid form, which does not require any involvement from the host immune system. This putative mechanism is orthogonal to many other anti-coronavirus agents that are in development [12].

**Figure 3.**
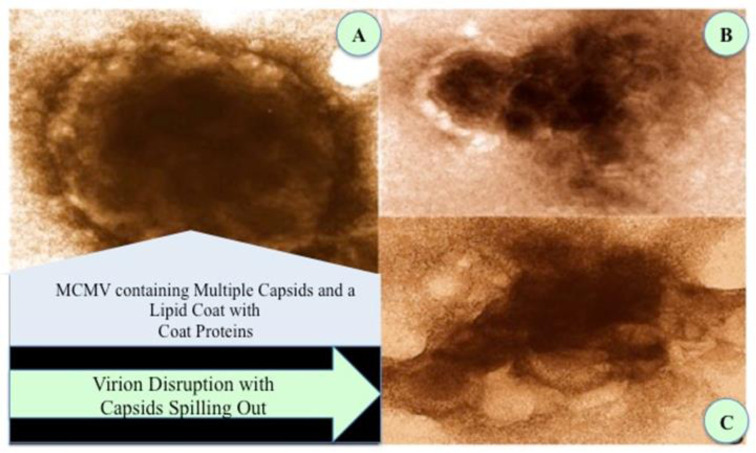
The effects of two different nanoviricides binding to Murine Cytomegalovirus (MCMV). A: Control Treated virion: MCMV containing multiple capsids and a lipid coat with coat proteins; B, C: MCMV virions treated with two different nanoviricides. Virion disruption with capsids spilling out.

The support of this mechanism is shown in the electron photomicrographs of Figure 3. In this study, the murine cytomegalovirus (CMV) was incubated with a nanoviricide. The loss of the viral envelope occurred when the nanoviricide started to bind to the CMV receptors, which made the virus non-infectious to other cells (Figure 3B,C). As a result, the binding of the nanoviricide renders the CMV to become inactive due to the capsid organization becoming disrupted. The proposed mechanism of action for the nanoviricides is not expected to interfere with the intracellular replication of the virus to any appreciable extent [13]. Therefore, it may be possible to achieve a stronger antiviral effect in the future by combining NV-CoV-2 therapy with other anti-coronavirus therapies.

### 2.3. Protection by encapsulation during delivery of other antiviral drugs

It's possible that NV-CoV-2 can only deliver encapsulated cargo into infected cells and not healthy cells. In fact, it's only the infected cells that display the viral antigen S-protein (S1 and S2 proteins) on either the budding virion or on the exposed cell membrane structures were NV-CoV-2 binds. In addition, NV-CoV-2 can encapsulate to protect other antiviral drugs and act as a double arm effector in humans.

It is hypothesized that NV-CoV-2 may provide substantial antiviral effects *in vivo*, which could result in rapid recoveries of the patients. The proposed combination is NV-CoV-2 encapsulating remdesivir (RDV) during the standard RDV treatment. RDV is the only antiviral drug approved and being used against the SARS-CoV-2 virus. RDV is the only anti viral that has recently been approved for using against SARS-CoV-2 virus. However, the efficacy of RDV *in vivo* studies is very low compared to *in vitro* cell culture studies. RDV is very unstable in the presence of plasma [2]. However, when RDV was encapsulated by NV-CoV-2, the antiviral efficacy of RDV increased in the rat studies [3],[4]. In this study the untreated rats and the vehicle-treated rats infected with the CoV-NL63 virus only survived for 5 days. The rats treated daily with a dose of RDV at 10 mg/kg, survived up to 7.5 days. The group of rats treated with NV-CoV-2, 320 mg/kg survived until day, 14. The survival rate of the rats administered with NV-CoV-2-R for 5 days increased to 18 days [3]. Therefore, NV-CoV-2-R clearly demonstrates antiviral efficacy in this *in vivo* animal study infected with the CoV-NL63 virus.

## 3. Discussion

NV-CoV-2 is a “nanoviricide^®^” that is designed to attack most strains of coronaviruses in a broad-spectrum manner. It is composed of a number of antiviral small chemical ligands that are covalently bound to a micelle-forming polymeric chain. This particular ligand is designed through molecular modeling to bind to the S-protein of SARS-CoV-1 and prevent the virus from binding to the cognate cellular receptor, ACE2. Therefore, NV-CoV-2 was designed to prevent coronaviruses such as SARS-CoV-1, SARS-CoV-2 and NL63 from binding to the ACE2 receptor. NV-CoV-2 also has activity against the human coronavirus 229E (hCoV-229E), which binds to the Aminopeptidase N (APN). This appears to be because of the certain conserved elements in the quaternary structures of ACE2 and APN that are both peptidases with different specificities. The broad-spectrum activity of NV-CoV-2 against both h-CoV-NL63 and h-CoV-229E indicates that it may continue to be active against a number of coronaviruses, even after it mutates [1].

The mechanism of action of NV-CoV-2 is not well defined yet, but is hypothesized to bind to free virion particles at multiple points and encapsulate the virus. Once the virus is encapsulated, it becomes neutralized and its ability to infect cells is disabled. This putative mechanism is orthogonal to many other anti-coronavirus agents that are being developed. Furthermore, it may be beneficial and possible to achieve a stronger antiviral effect by combining NV-CoV-2 nanoviricide with other anti-coronavirus therapies in the future.

We have experienced that NV-CoV-2 works in three different ways to destroy the SARS-CoV-2 virus. which causes COVID-19. First, a nanoviricide micelle will bind to a virus particle because of the specific interaction between a ligand on the nanoviricide with the glycoproteins on the virus surface. The flexible nanoviricide can get close to the virus and cause additional ligands to bind to additional viral coat proteins, in a mode called “co-operative binding”. Co-operative binding is a well-known natural process that forms the basis of biological recognition such as antibody-antigen binding complexes, DNA hybridization, and protein assembly. The idea that multiple ligands are attached to a single polymer chain and multiple polymer chains make up a single micelle may lead to a very high avidity for the micelle to bind to the virus. This is analogous to the “Velcro” effect that results from cooperative interactions. This property enables the use of ligands that may individually have low affinity, but the polyvalency can result in very high avidity. This concept has been shown quite convincingly with polymeric vs. monomeric inhibitors in the scientific literature [12],[13].

An analogy may be drawn between nanoviricides and neutralizing anti-viral antibodies. Antibodies are an integral part of the natural immune process to bring infections under control. Antibodies can ameliorate or prevent many viral infections and may aid in the resolution of viral infections. Some antibodies do this through a process called neutralization. Antibodies can neutralize viral infectivity by causing aggregation of the virus particles, inhibiting the virus from binding to its host cell surface receptor(s), blocking endocytic uptake into cells and/or preventing virus uncoating in endosomes. When anti-viral antibodies bind to the viruses, the resulting complexes can be lysed upon subsequent binding by complement factors. In addition, virus-antibody complexes can be taken up by phagocytes and destroyed. In contrast, a nanoviricide micelle binds to a virus particle through van der Waals interactions and promotes encapsulation of the virus particle. Re-arrangement of the polymeric chains takes place where the lipid tails of the nanoviricide micelle integrate with the lipid membrane coating the virus (for “enveloped” viruses). Encapsulation of the virus particle may lead to the viral envelope becoming dismantled, viral surface lipids getting stripped off and the removal of glycoproteins that are necessary for viral adsorption and cellular entry.

Viruses bind to specific cell surface ligands in order to attach to cells and internalize. Nanoviricides become very useful by neutralizing these crucial steps of the viral particles. A nanoviricide is designed to act like a human cell decoy. When the virus sees the appropriate ligand displayed on a nanoviricide micelle, the virus is believed to bind to it. The nanoviricide is very flexible and can bind all over the viral particle surface, which could potentially lead to fusion with the lipid-coated virus surface by a phase-inversion. Basically, the fatty core of the nanoviricide could merge with the viral lipid coat and the hydrophilic shell of the nanoviricide could become the exterior of the particle, completely engulfing the virus. Once encapsulation occurs the coat proteins that are necessary for the virus to bind to other cells becomes unavailable. This highly targeted attack may also lead to a loss of viral coat proteins and the nanoviricide may further dismantle the engulfed virus capsid. The loss of virus particle integrity could render the virus non-infectious. This proposed mechanism of action (Figure 2) supports the electron photomicrographs (Figure 3), where the murine cytomegalovirus (CMV) was incubated with a nanoviricide. Figure 3B,C demonstrates inactivity because it is believed there is a disruption of capsid organization. Given this proposed mechanism of action, nanoviricides are not expected to interfere with the intracellular replication of the virus to any appreciable extent.

**Figure 4.**
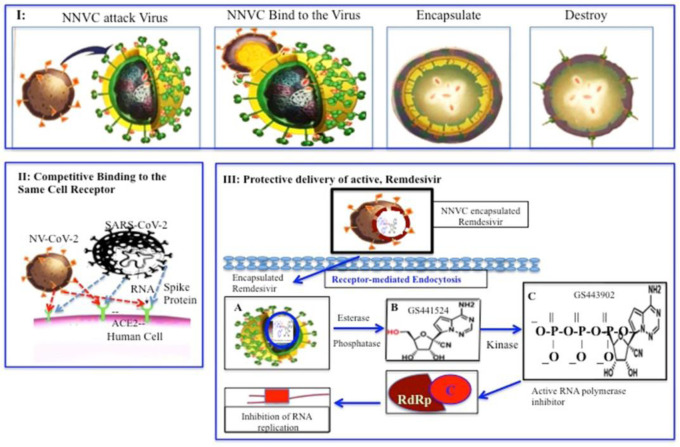
Graphical representation of a *Nanoviricide* (NV-CoV-2) mechanism of action. Mechanism I: Step I–1: NNVC attacks Virus; Step I–2: NNVC binds to the Virus; Step I–3: Encapsulate the Virus; Step I–4: Destroy the Virus. Mechanism II: Competitive binding of Virus and *Nanoviricide* to the same cell receptor. Mechansim III: Protective delivery of active antiviral RDV by *Nanoviricide* enabling a double arm effect against virus.

The antiviral efficacy, potency and the cytotoxicity of the antiviral compound NV-CoV-2 was studied in cell cultures [4]–[8]. A relative CPE level was compared between all groups to identify any reduction in the viral replication. Reduction in the CPE levels was compared to untreated infected controls which suggests there is a decrease in the virus production, growth and spread. Furthermore, the polymer encapsulation renders RDV highly effective against SARS-CoV-2 by protecting the drug against the plasma [4] (Figure 4).

Treatment with NV-CoV-2 and NV-CoV-2-R extended the survival of the rats infected intra-tracheally with a lethal dose of human CoV-NL63 virus. In fact, NV-CoV-2-R showed a better effect than NV-CoV-2, and both treatments were better than RDV treatment alone. Thus, NV-CoV-2-R clearly demonstrates antiviral efficacy in this lethal infection model with CoV-NL63 virus.
